# miR-29a/b Enhances Cell Migration and Invasion in Nasopharyngeal Carcinoma Progression by Regulating SPARC and COL3A1 Gene Expression

**DOI:** 10.1371/journal.pone.0120969

**Published:** 2015-03-18

**Authors:** Feifei Qiu, Rui Sun, Ning Deng, Tianyu Guo, Yange Cao, Ying Yu, Xuejun Wang, Bingcheng Zou, Songmei Zhang, Tao Jing, Tao Ling, Jun Xie, Qing Zhang

**Affiliations:** 1 State Key Laboratory of Biocontrol, School of Life Sciences, Sun Yat-sen University, Guangzhou, China; 2 State Key Laboratory of Oncology in Southern China; Collaborative Innovation Center for Cancer Medicine, Sun Yat-sen University Cancer Center, Guangzhou, China; 3 Key Laboratory of Molecular Immunology and Antibody Engineering of Guangdong Province, Antibody Engineering Center in Jinan University, Guangzhou, China; 4 Key Laboratory of Tropical & Subtropical Fishery Resource Application & Cultivation, Ministry of Agriculture, Pearl River Fisheries Research Institute of CAFS, Guangzhou, Guangdong, China; New England Biolabs, Inc., UNITED STATES

## Abstract

Nasopharyngeal carcinoma (NPC) is a malignant tumor associated with a genetic predisposition, Epstein-Barr virus infection and chromosomal abnormalities. Recently, several miRNAs have been shown to target specific mRNAs to regulate NPC development and progression. However, the involvement of miRNAs in processes leading to NPC migration and invasion remains to be elucidated. We predicted that miR-29a/b are associated with dysregulated genes controlling NPC through an integrated interaction network of miRNAs and genes. miR-29a/b over-expression in NPC cell lines had no significant effect on proliferation, whereas miR-29b mildly increased the percentage of cells in the G1 phase with a concomitant decrease in the percentage of cells in S phase. Furthermore, we demonstrated that miR-29a/b might be responsible for increasing S18 cell migration and invasion, and only COL3A1 was identified as a direct target of miR-29b despite the fact that both SPARC and COL3A1 were inhibited by miR-29a/b over-expression. Meanwhile, SPARC proteins were increased in metastatic NPC tissue and are involved in NPC progression. Unexpectedly, we identified that miRNA-29b expression was elevated in the serum of NPC patients with a high risk of metastasis. The 5-year actuarial overall survival rates in NPC patients with high serum miR-29b expression was significantly shorter than those with low serum miR-29b expression; therefore, serum miR-29b expression could be a promising prognostic marker.

## Introduction

NPC is one of the most prevalent malignancies of the head and neck in southern China, with a high incidence rate of approximately 10–50/10^5^ individuals per year [1, 2]. The geographic distribution of NPC indicates its unusual etiology. Three major etiologic factors, genetic, environmental, and viral, have been identified as leading to multiple genetic and epigenetic alterations during NPC pathogenesis by either acting alone or in synergy [3]. Although certain oncogenes and tumor suppressor genes play important roles in NPC pathogenesis, a complete understanding of the pathogenesis of NPC in the context of global gene expression, transcriptional pathways and biomarker assessment remains to be elucidated. Fortunately, certain small non-coding RNAs have recently emerged as master regulators of NPC gene expression by targeting protein-coding mRNAs.

miRNAs have been shown to be important gene regulators in many organisms and have already been implicated in a growing number of diseases. The aberrant expression of miRNAs in different NPC stages suggests that they may have a critical role in the coordinate regulation on target gene expression. Several miRNAs have been shown to target specific mRNAs to regulate NPC development and progression. miRNAs of the let-7 family suppress NPC cell proliferation by down-regulating c-Myc expression [4]. However, those studies did not offer a fully comprehensive view of miRNA-dependent regulation of NPC genes. The availability of rapid and accurate bioinformatic methods and the development of efficient algorithms have provided a high level of confidence in miRNA predictions. Evidence has shown that microRNAs suppress their target mRNAs by imperfect base pairing with their 3′ untranslated region (3′-UTR) [5, 6]. In a search to match the miRNA: mRNA pairs in the large number of potential targets with conventional 3′-UTR sites, prioritizing searches using miRanda and TargetScancan expedite target identification. Additionally, because miRNAs could directly interact with their target genes and affect the expression of many other genes indirectly, changes in non-target mRNAs may be discernible in the transcriptional profile when an miRNA was aberrantly expressed. Thus, with the increased use of miRNA microarrays and transcriptome expression data, systematic investigation on the interactions between target genes and miRNAs could yield more accurate information on miRNA regulation [7–9]. The miRNAs expression pattern showed that they were highly differentially expressed, with specific miRNAs active in certain tissues during certain times. In many cancers, miRNA expression was significantly altered and could contribute to cancer development and progression. Among these miRNAs were species with a well-characterized cancer association, such as the over-expressed miR-21 and the under-expressed miR-29c [10, 11]. The predicted targets for the differentially expressed miRNAs are significantly enriched for protein-coding tumor suppressors and oncogenes. Thus, the functional significance of miRNA dysregulation may serve to help identify and characterize tumors in human tissues.

The miR-29 family has emerged in various tissues as a key modulator of extracellular matrix (ECM) homeostasis. The enforced expression of miR-29 induced apoptosis in cancer cell lines and reduced tumorigenicity [12]. These profound tumor suppressor effects could be partly explained by the direct targeting of apoptosis-associated factors and extracellular matrix proteins by the miR-29 family [10, 13]. Conversely, miR-29 family members have been shown to be downregulated in CLL, lung cancer, invasive breast cancer, AML, and cholangiocarcinoma [12]. There are three members in the human miR-29 family: miR-29a; miR-29b; and miR-29c. miR-29a not only acted as a tumor suppressor by regulating its target genes Tcl1 and DNMT3 in chronic lymphocytic leukemia and lung cancer [14] but also up-regulated p53 levels and induced apoptosis in a p53-dependent manner [15]. However, it was reported that miR-29a promoted tumorigenesis in breast cancer, as over-expression of miR-29a led to epithelial-to-mesenchymal transition and metastasis in cooperation with oncogenic Ras signaling [16]. Meanwhile, miR-29b was found to be a positive regulator of osteoblast differentiation by down-regulating inhibitory factors of osteogenic signaling pathways and controlling expression of collagen in differentiated osteoblasts [17]. Cortez et al. demonstrated that miR-29b directly targeted the 3′-UTR region of PDPN and inhibited invasion, apoptosis, and proliferation of glioblastomas [18]. miR-29b has also played an epigenetic role in targeting expression of DNA methyltransferases (DNMT3A and -3B) in multiple myelomas, resulting in significant anti-tumor effects [19]. Of note, most miR-29c-targeted genes encode extracellular matrix proteins that were associated with tumor cell invasiveness and metastatic potential, prominent characteristics of NPC [10]. This evidence suggested a context-dependent pattern for the miR-29 family in tumorigenicity.

Metastasis proceeds through the progressive acquisition of traits that allow malignant cells originating in one organ to disseminate and colonize secondary site. This process could involve the concomitant recruitment of miRNAs that are advantageous to cancer cells. Most extracellular matrix (ECM) components and extracellular matrix regulators have a multi-domain structure in which individual modules have specific functions in the modulation of cell–cell, cell–matrix interactions or supramolecular assembly [20, 21]. Indeed, miR-29c was first found to be down-regulated in NPC and involved in metastasis by regulating mRNAs identified by encoding ECM proteins, such as secreted protein acidic and rich in cysteine (SPARC) and COL3A1[10]. SPARC has been correlated with metastasis based on changes in cell shape, which could promote tumor cell invasion. In contrast, it has also been associated with tumor suppression because it could decrease the mitogenic potency of various growth factors by antagonizing their ability to bind to their cognate receptors [22, 23]. Of equal import might be the effect of COL3A1 on ECM production and assembly, which is found in extensible connective tissues, is also an extracellular matrix component involved in cell migration and metastasis. ECM reorganization may facilitate motility and migration during normal development, while tumor invasion may resemble dysregulated developmental processes at the tumor-stromal interface through spreading into neighboring ECM environments [24, 25]. In particular, regulatory effects of SPARC on COL3A1 in extracellular matrix components have been observed in normal cultured human fibroblasts, in which, both SPARC and COL3A1 were involved in the regulation of collagen expression as well as the regulation of each other. Increased SPARC expression may contribute to its regulation of downstream genes, including COL3A1; this situation appeared to play a protective role against profibrotic over-expression of collagen genes. However, it was still unclear whether the excessive deposition of ECM in NPC cells would be associated with SPARC and other related factors.

In the present study, we used not only microarray datasets to analyze the mRNA expression profile of tumors from NPC patients and normal nasopharyngeal tissue but also an integrated method to predict miRNAs targeting dysregulated mRNA in NPC. Then, we constructed the association between endogenous mRNA expression and miRNAs, which subsequently permitted us to identify the potentially functional miR-29a and miR-29b. Studies of gain- and loss-of-function of miR-29a/b were used to validate the prediction into the extent of their influence in NPC cell lines. Furthermore, we detected whether SPARC and COL3A1 could be identified as targets of miR-29a/b in NPC cell lines. We then analyzed the possible mechanisms by which SPARC and COL3A13 promoted NPC cell migration and invasion. Of note, we found that miR-29b has potential function in screening serum biomarkers in NPC patients with a high risk of metastasis, thus highlighting the significance of miR-29a/b in NPC tumorigenesis.

## Materials and Methods

### Selection of genes related to NPC and construction of interaction network

We searched Gene Expression Omnibus (GEO) for the entire human gene expression profile of NPC. The dataset (GSE12452) with 31 different NPC samples and 10 normal reference samples analyzed by Affymetrix array was utilized in our study. The genes with expression that changed at least 4-fold were chosen as our research focus. We searched the BioGRID database for genetic interactionsin NPC and downloaded available gene data in the Osprey Custom Network file format. Then, we constructed an interactive network of both up- and down-regulated genes.

### Prediction of miRNA-targeting genes

For prediction of miRNA-targeting genes with altered expression in NPC, miRanda, TargetScan and MicroCosm Target software was employed. Conservation criterion was not utilized, as it was found that the nonconserved sites could also contribute to repression. miRNAs predicted by those three programs for every gene were assayed, and the overlapping results were extracted for subsequent analysis.

### Construction of miR-29a/b expression and luciferase reporter plasmids

To construct a plasmid expressing miR-29a/b, we amplified a 150–500 bp DNA fragment containing a miR-29a/b precursor from human genomic DNA (293T) and cloned the amplified fragment into a modified pEGFP-C1 (Clontech), generating pEGFP-miR-29a/b. TargetScan 4.1 (http://www.targetscan.org), a miRNA target prediction program, was used to search for putative miR-29a/b targets. A 580-bp fragment from the 3′-UTR of wild-type (WT) SPARC and a 510-bp fragment from the 3′-UTR of wild-type COL3A1 containing the miR-29a/b-binding sites were cloned into the psiCHECK-2 vector (Promega) downstream of the Renilla luciferase gene (Xho I/Not I sites). The mutant-type (MT) construct was identical to the WT construct, except that it had three point substitutions disrupting pairing to each miR-29a/b. Mutant plasmids, in which the mutated regions were complementary to seed regions of miR-29a/b binding, were also constructed.

### Cell culture and luciferase assay

The human 293T line was obtained from American Type Culture Collection (http://www.atcc.org); The human NPC S18 cell line was isolated from the parental line CNE-2 by limiting dilution method, as previously described [26]. The human 293T line cell line and NPC S18 were maintained as adherent cultures in Dulbecco’s Modified Eagle’s Medium (DMEM) supplemented with 10% fetal bovine serum (FBS; Gibco), 100 U/mL penicillin, and 100 U/mL streptomycin and were incubated at 37°C in a humidified chamber supplemented with 5% CO2. 293T cells were seeded into 48-well plates (6.0 × 10^4^ per well). After 24 h, the cells were cotransfected with the reporter vectors and the miRNA-expressing plasmid (pEGFP-miR-29a/b or pEGFP-control) as well as miR-29a/b inhibitors (Ribobio) at a ratio of 0.1 μg: 0.1 μg using Lipofectamine 2000 (Invitrogen). Luciferase activity was measured 48 h post-transfection using the Dual-Luciferase Reporter Assay System, according to the manufacturer’s instructions (Promega). For each sample, Renilla luciferase activity was normalized to firefly luciferase expression.

### MTT [3-(4,5-dimethylthiazol-2-yl)-2,5-diphenyltetrazolium bromide] assay

S18 cells (5×10^3^) were seeded into 96-well plates. MTT (Sigma-Aldrich) assays were performed after transient transfection with pEGFP-miRNA. At different time points, the medium was replaced with fresh medium containing 0.5 mg/ml MTT for 4 h at 37°C, and then, the MTT was carefully removed. Dimethyl sulfoxide (150 μl) was added to each well to dissolve the formazan crystal, and the optical density at 492 nm was determined using a multifunctional microplate reader (TECAN Infinite M200, Switzerland).

### Flow cytometry analysis

After transfection for 48 h, the cells were harvested and washed with PBS twice and fixed with 70% ethanol overnight at −20°C. The cells were washed with PBS again, stained with 5 μl of propidium iodide solution (10 μg/ml) and 100 μl of RNase (100 μg/ml) in PBS and incubated for 30 min at room temperature. The analysis was performed using FACS Calibur (Becton Dickinson, Franklin Lakes, NJ) and CellQuest Pro software (Becton Dickenson).

### Transwell migration and invasion assay

Cell migration was measured using a transwell migration assay that was performed using an 8-μm pore size transwell chamber (BD Biosciences, Bedford, MA). S18 cells were transfected with pEGFP-miR-29a/b or miRNA inhibitors as mentioned above. At 24 h post-transfection, cells (5×10^4^ in 500 μl of blank medium) were reseeded into the rehydrated insert. Medium with 5% FBS was added to the lower chamber as attractant. After a 24-h incubation, non-invading cells on the upper surface of the membrane were scrubbed. The migrated cells that pushed themselves through the pore and grew on the lower surface were fixed with 100% methanol and stained with 0.1% gentian violet. The stained invasive cells were imaged and quantified by manual counting in three randomly selected areas. Cell invasion was measured by a Matrigel invasion assay, which was performed using BD Matrigel Invasion Chambers (BD Biosciences, Bedford, MA).

### Western blotting

Cells were transfected with 50 nM negative control RNA mimics (denoted as NC), mimics-miR-29a/b, anti-scramble (control anti-miRNA) and anti-miR-29a/b siRNA duplexes for SPARC and COL3A1 (Gene Pharma) in 24-well plates. Cell samples and nuclear/cytoplasmic extracts were prepared according to the manufacturer’s instructions (Thermo), collected 48 h later, and analyzed using Western blotting. GAPDH (Cell Signaling Technology) was used as a loading control. Protein expression was detected by incubation with either rabbit polyclonal anti-SPARC or anti-COLA1 (Santa Cruz Biotechnology). Immunoreactive bands were detected by ECL (Amersham, USA) using horseradish peroxidase-labeled secondary antibodies (Cell Signaling Technology).

### Patient samples and real-time quantitative RT-PCR

The consecutive NPC patients who were newly diagnosed between July 2011 and August 2012 were recruited from Sun Yat-Sen University Cancer Center for this study. This study included 193 patients and 65 healthy donors from the Nasopharyngeal Carcinoma Department of Sun Yat-Sen University Cancer Center. Of these patients, we identified 110 NPC patients with low metastatic/invasive cancer and 83 NPC patients with high metastatic/invasive cancer who had completed radical treatment during the study period. Written informed consent was obtained from this study participants. This study was approved by the Clinical Ethics Review Board of Sun Yat-Sen University Cancer Center. The collection and use of tissues followed the procedures that are in accordance with the ethical standards as formulated in the Helsinki Declaration. The patient eligibility criteria were as follows: an age of 18–65 years, pathological confirmation of undifferentiated non-keratinized or differentiated non-keratinized carcinoma of the nasopharynx, a Union for International Cancer Control (UICC) staging system 2009 clinical classification of I to IVb. The exclusion criteria included a history of anticancer therapy, pregnancy or lactation, and the presence of contraindications for receiving chemotherapy or radiotherapy. Blood samples were obtained by venipuncture prior to anticancer therapy, centrifuged at 3000 rpm for 10 min and then frozen at −80°C until analysis. Serum miRNAs from these patients were isolated using TRIzol reagent. A total of 500 μl of TRIzol reagent and 100 fmol cel-miR-39 mimics (Ribobio) as a control were added into 500 μl of serum and incubated for 5 min; then, 200 μl of chloroform was added according to the TRIzol protocol. Quantitative RT-PCR assays were conducted to quantify mature miRNA expression using an SYBR Green PCR Master Mix (Toyobo) on a Bio-Rad iCycler iQ5 Detection system. The amount of miR-29a/b was normalized to cel-miR-39 and calculated according to the comparative cycle threshold (Ct) method. The miR-29a primers were as follows: reverse transcription primer, CTCAACTGGTGTCGTGGAGTCGGCAATTCAGTTGAGTAACCGAT; forward primer, CCGTCCTCCGTAGCACCATCTGAAAT; and reverse primer, CTCAACTGGTGTCGTGGAGTCGGC. The miR-29b primers were as follows: reverse transcription primer, CTCAACTGGTGTCGTGGAGTCGGCAATTCAGTTGAGAACACTGA; forward primer, CGCTCCTCCGTAGCACCATTTGAAATC; reverse primer, CTCAACTGGTGTCGTGGAGTCGGC. All reactions were run in triplicate.

### Immunohistochemistry analysis

The clinically stratified NPC tissue samples used in this study have been described above. All the diagnoses were confirmed by pathology. Written informed consent was obtained from each patient participating in this study. All of the protocols were reviewed and approved by the Ethics Committee of Clinical Ethics Review Board of Sun Yat-Sen University Cancer Center and performed in accordance with national guidelines. SPARC and COL3A1 expression was evaluated on a wide range of NPC samples to determine the intensity and extent of expression in tissue sections. Immunohistochemistry was performed on the samples of benign NPC histologically diagnosed, non-disseminated NPC, low metastatic/invasive samples and high metastatic/invasive samples using standard biotin-avidin complex analysis. Protein expression was scored as negative (score = 1), weak (2), moderate (3) and strong (4) by anti-SPARC or anti-COL3A1 (Cell Signaling Technology) staining assessment. Four replicate tissue cores were sampled from each of the selected tissue types.

### Statistical Analyses

Variables were compared using the t-test or a one-way ANOVA, whenever appropriate. Statistical significance was considered at a *p* value < 0.05. All analyses were performed using the Statistical Package for the Social Sciences (SPSS) (IBM Corp. Released 2011. IBM SPSS Statistics for Windows, Version 20.0. Armonk, NY: IBM Corp). The mean ± SD is displayed in the figures. The survival probabilities were determined using Kaplan-Meier analysis, and the significance of differences was analyzed by the log-rank test.

## Results

### Integrated analyses set association with gene expression alterations and miRNAs

**Fig 1:**
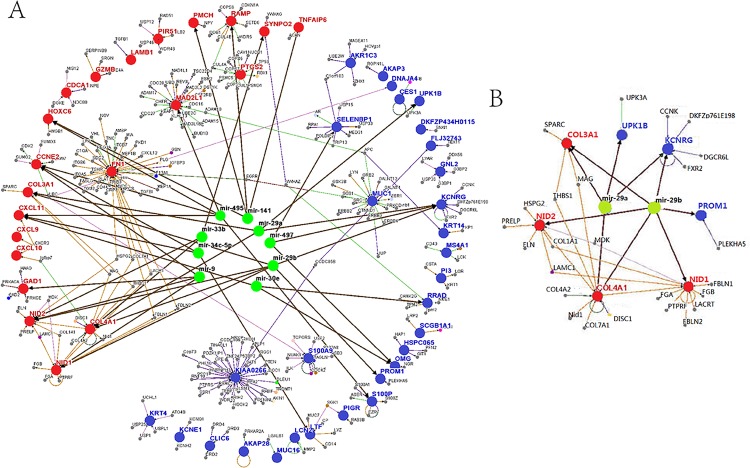
Integrated microRNA-gene network. (A) The miRNA-gene network shows the relationships between 9 miRNAs and 51 dysregulated genes. Only genes reported in BioGrid are shown in this network. Interaction among genes and regulation of miRNAs are indicated by arrows. Red nodes represent up-regulated genes in NPC, and blue nodes represent down-regulated genes. miRNAs with more than 2 targets are shown in the figure. (B) The particular network for miR-29a/b and their targets stems from Fig. 1A.

The interplay between microRNAs and their target genes contributes to cancer development and progression, and miRNAs are differentially expressed in normal tissues and cancers. We speculated that combining mRNA expression with related alteration in miRNA systems would help identify molecular driver events. Here, we selected 171 human NPC-related genes whose expression changed at least 4-fold from the microarray dataset GSE12452 (from NCBI GEO), including 32 up-regulated genes and 139 down-regulated genes. Subsequently, protein interactions of dysregulated genes with other genes were obtained from the BioGRID database. This analysis narrowed the list to 51 genes which contained 21 up-regulated genes and 30 down-regulated genes. Some selected genes were not reposited by BioRGID, aiding to refine the comprehensive interaction network and interrogate the function of proteins encoded by dysregulated genes. Furthermore, predictions of miRNAs using bioinformatic methods, including TargetScan, miRanda and MicroCosm Target, were applied to genes presented in the network. A set of 9 miRNAs was predicted to target multiple genes. We first identified this master miRNA regulatory network for targeting multiple genes in NPC using an integrated network analysis (Fig. 1A). The gene-associated miR-29a and miR-29b, found to be dysregulated in NPC [27, 28], were among the 9 identified miRNAs and predicted to target a set of up-regulated genes, including COL3A1, COL4A1, NID1 and NID2 (Fig. 1B) together with their downstream genes. The predicted regulatory targets of miRNA29a/b were enriched for genes involved in extracellular matrix regulation but also encompassed an unexpectedly broad range of other functions.

### SPARC and COL3A1 were inhibited by miR-29a/b over-expression

**Fig 2:**
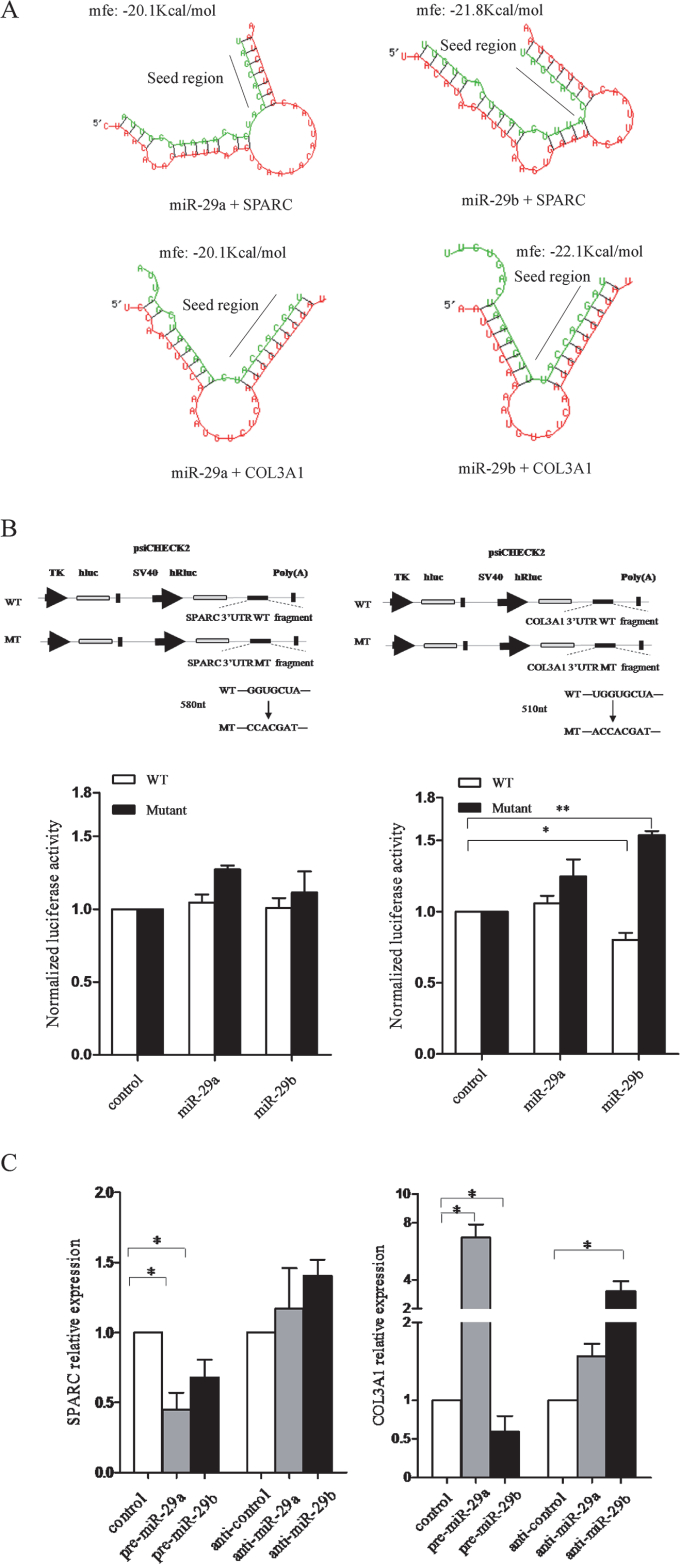
Identification of SPARC and COL3A1 as miR-29a/b targets. (A) Conserved miR-29a/b binding sites in the 3′-UTRs of SPARC and COL3A1 are predicted by TargetScan; candidates were filtered using a hybridization free-energy threshold of −19.0 (kcal/mol).The sequences in green refer to miRNA, and the sequences in red refer to the 3′-UTR of mRNA. (B) A schematic diagram showing the 3′-UTR reporter constructs. The sequences of the wild type or mutant site in the 3′-UTR fragments are shown. MT: Nucleotide substitutions disrupting the miRNA-29a/b-binding sites were introduced in the 3′ UTRs of SPARC and COL3A1 cloned downstream of the Renilla luciferase gene. (C) Luciferase activities were measured 48 h after transfection with plasmids pEGFP-miR-29a/b and their mutants. The activities of Renilla luciferase were normalized to firefly luciferase activities, and the target validation data were confirmed in duplicate experiments. (D) Quantitative RT-PCR of SPARC and COL3A1 expression in S18 cells after transfection of miR-29a/b and their inhibitors for 48 h. *p < 0.05.

The finding that miR-29a/b regulate certain genes involved in the extracellular matrix allowed us to hypothesized that miR-29a/b may post-transcriptionally affect NPC cell mobility. Among the predicted genes, we were particularly interested in COL3A1 and SPARC, which have been reported to regulate NPC carcinogenesis [29], both of which act as potential functional targets of the miR-29 family[20, 30, 31]. To validate the prediction in NPC, we identified a putative consensus site for miR-29a/b binding in the 3′-UTR of COL3A1 and SPARC by TargetScan (release 4.1; http://www.targetscan.org). The 3′-UTR segments harboring the WT or MT candidate motifs targeted by miR-29a and miR-29b were synthesized and subcloned downstream from the reporter gene in the psiCHECK-2 vector (Fig. 2A and B). Subsequently, we applied experimental support for the predicted targets. Luciferase reporter constructs containing either a wild-type (COL3A1/SPARC) or mutated 3′-UTR were co-transfected with pre-miR-29a/b into HEK293T cells. Reporter assays revealed that miR-29b specifically suppressed the luciferase activity driven by the 3′-UTR of COL3A1 mRNA, and mutating the miR-29b target sites in the 3′-UTR abrogated miR-29b-induced inhibition of luciferase expression, demonstrating that mRNA of COL3A1 is a direct target of miR-29b. In contrast, miR-29a had no appreciable effect on luciferase activity driven by the 3′-UTR fragment of COL3A1 mRNA, despite the fact that it has a putative binding site for miR-29a (Fig. 2B). Meanwhile, we detected the mRNA level of both targets in response to the expression change of miR-29a and miR-29b. We found that over-expression of miR-29a and miR-29b significantly down-regulated SPARC expression by real-time PCR in the S18 cell line, whereas knockdown of both miR-29a and miR-29b insignificantly up-regulated SPARC. Conversely, the up-regulation of miR-29a significantly increased COL3A1 expression, while only miR-29b expression was negatively associated with COL3A1 expression (Fig. 2C).

### miR-29b inhibited cell cycle progression at the G1/S transition without affecting cell proliferation

**Fig 3:**
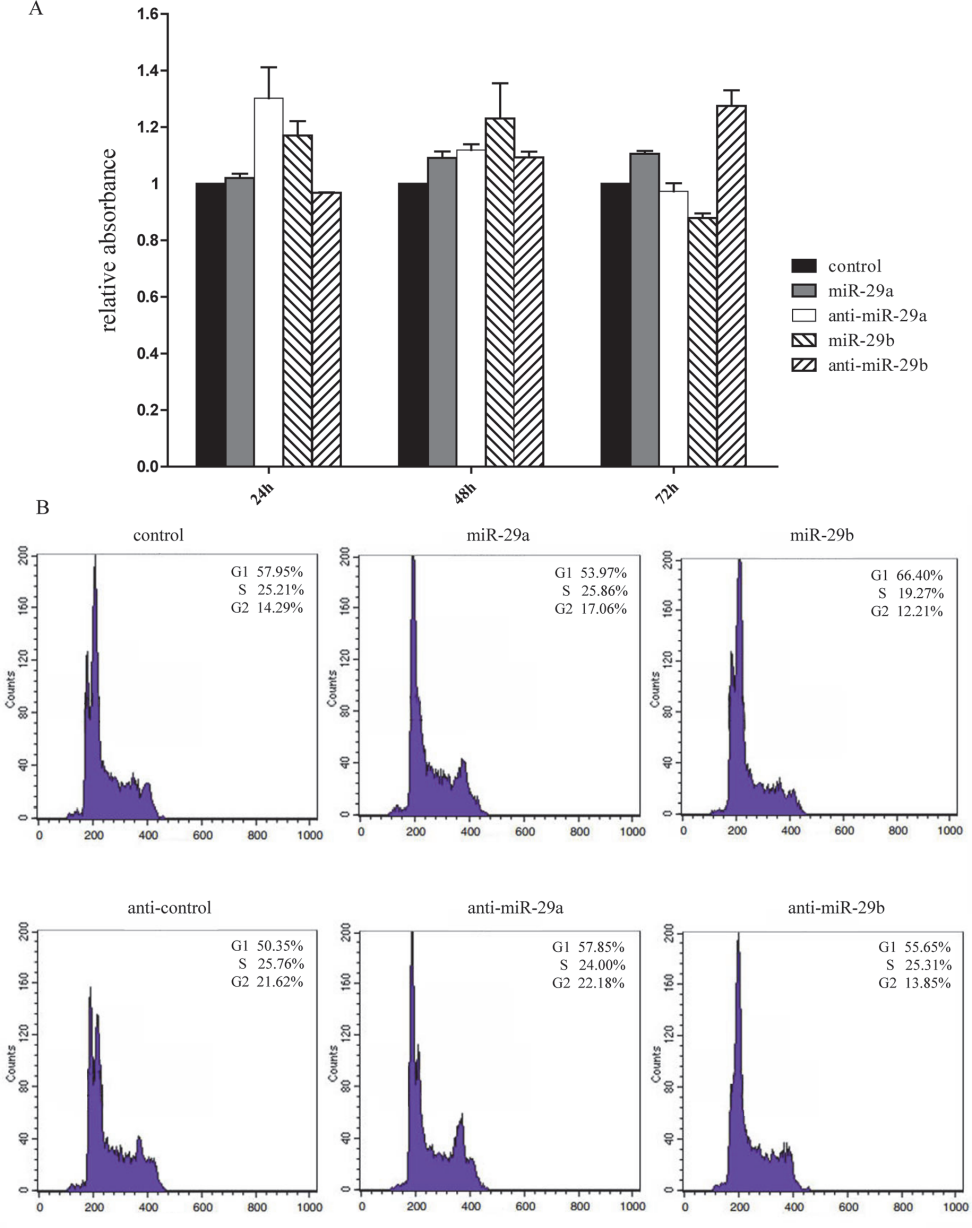
Effects of miR-29a/b on NPC cell growth and cell cycle. The results of MTT assays following transfection of pre-miR-29a/b and their inhibitors into S18 cells for the indicated 24 h, 48 h and 72 h post-transfection times. The values are the mean and SD optical density (OD) units. (B) miR-29b increased the proportion of S18 cells at the G1/S transition, whereas miR-29a did not have a similar effect. Cells were treated with pEGFP-miR-29a/b or anti-miR-29a/b transfection and control vector pEGPF-C. Cell cycle distributions were detected 20 h later. A representative result of 3 independent experiments is shown. In all experiments, the negative control was pre-miRNA negative control.

Accumulating evidence suggests roles for miRNAs in human carcinogenesis as novel types of tumor suppressors or oncogenes [32]. We therefore explored the roles of miR-29a/b in tumorigenesis through an MTT assay and cell cycle analysis. S18 cells and highly metastatic NPC cell lines expressing low levels of miR-29a/b were chosen (data not shown). S18 cells over-expressing miR-29a/b were generated by transfecting pEGFP-miR-29a to over-express miRNAs along with their inhibitors to knockdown their expression. An MTT assay revealed no significant induction of proliferation in S18 cells by either miR-29a or miR-29b over different time periods (Fig. 3A), which indicates that neither miR-29a nor miR-29b mainly drives S18 cell proliferation. Further analysis of cell cycle progression by flow cytometry indicated that over-expression of miR-29b resulted in a mild increase in the G1 phase in S18 (proportion percentage from 57.95%–66.4%) and a concomitant decrease in the S phase (from 25.21% to 19.27%) compared with the control group. In contrast, little effect of miR-29a was noticed with regard to the cycle cell progression of S18 cells (Fig. 3B).

### miR-29a/b regulate NPC cells migration and invasion

**Fig 4:**
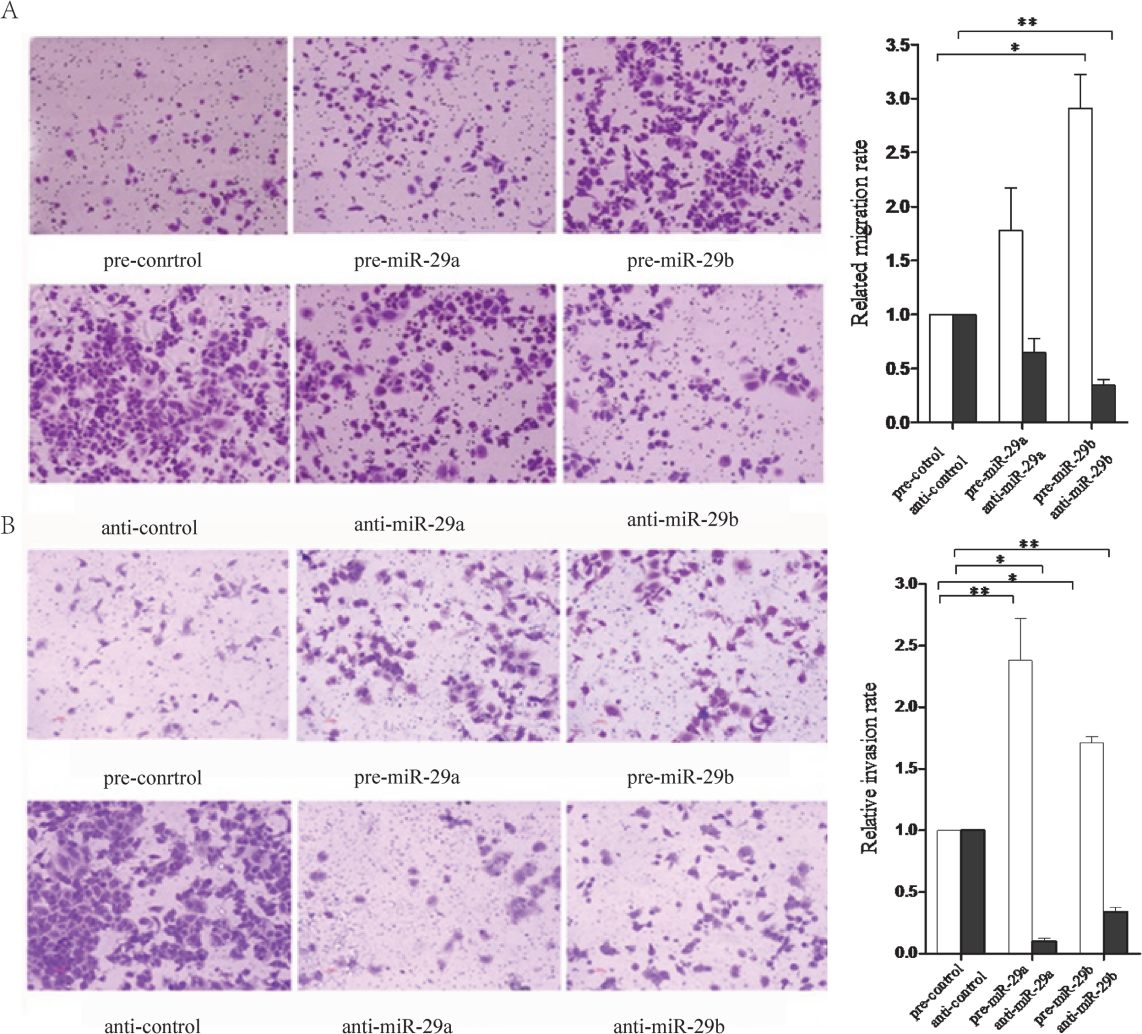
Effects of miR-29a/b expression on NPC cell migration and invasion. (A, B) Transwell migration and invasion assays showing the effect of miR-29a/b overexpression or knockdown on the migrated and invasive activity of S18 cells transfected with pEGFP-miR-29 or anti-miR-29. The migrated and invasive NPC cells that grew on the lower surface were stained and counted manually using a microscope (original magnification 50×) at 24 h after reseeding. Representative images are shown in the left panel. The mean number of cells per visual field was determined in four randomly selected visual fields per chamber and the experiments were performed in triplicate (right panel). *p < 0.05.

Oncogenic miRNAs are usually overexpressed in tumors or tumor cell lines and induce cell migration and invasion. In addition, suppressive miRNAs can be down-regulated, leading to tumor growth, carcinogenesis, and invasion, which depends on whether they specifically target oncogenes or tumor suppressor genes [33, 34]. To further test the hypothesis that tumor cells realign the collagenous matrix to facilitate local invasion by miR-29a/b, we next determined NPC cells migration and motility using transwell chamber assays. We found that tumor cell migration was significantly induced in S18 cells overexpressing miR-29a/b by 70% and 190%, respectively, compared with their respective control cells (*p* < 0.05), whereas knockdown of miR-29a/b prevented cell migration (Fig. 4A). In parallel, an invasion assay was performed to determine the effect on the invasion of S18 cells transfected with miR-29a/b, respectively. The results revealed that miR-29a/b increased S18 cells invasion by 130% and 70%, respectively, compared with NC, while inhibition of miR-29 clearly suppressed cell invasion (*p* < 0.05; Fig. 4B). These data suggest that miR-29a/b are potent regulators of S18 cell migration and invasion possibly through modulation of the matrix-related signaling pathway.

### miR-29a/b target the SPARC/COL3A1 pathways in NPC cells

**Fig 5:**
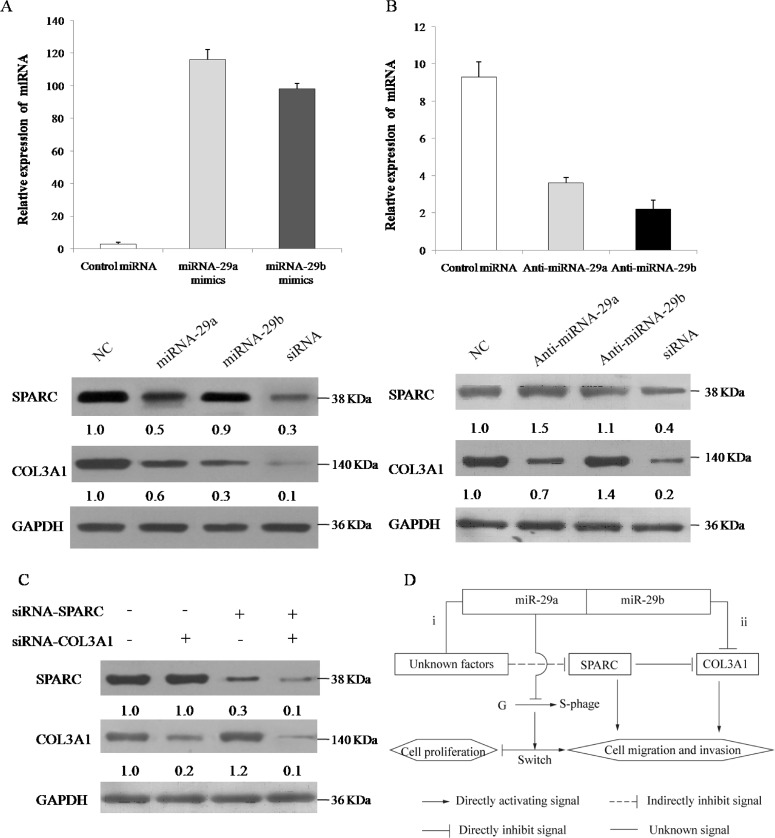
miR-29a/b target the SPARC/COL3A1 pathways in NPC cells. (A, B) S18 cells were transfected with 50 nmol of mimics-NC (control miRNA), mimics-miR-29a/b, anti-scramble (control anti-miRNA) and anti-miR-29a/b. The levels of miR-29a and miR-29b were assessed by qRT-PCR. Cell lysates were prepared for Western blotting with antibodies against SPARC and COL3A1, and GAPDH expression served as a loading control. Western blot figures are representative of at least three independent experiments. The value under each sample indicates the fold change of SPARC and COL3A1 protein levels relative to that of the control. (C) Western blot analysis of the expression level of SPARC and COL3A1 in NPC cells following treatment with vehicle, siRNA-SPARC and siRNA-COL3A1 for 24 h. The value under each sample indicates the fold changes of SPARC and COL3A1 protein levels relative to that of the control. Three independent experiments performed in triplicate. (D) A schematic model shows the function of miR-29a and miR-29b in NPC cell proliferation, migration and cell invasion. In response to miR-29a/b stimuli, the G1/S transition arrest triggers both a classical proliferative inhibition and an adapted metabolic switch. SPARC and COL3A1 protein levels inversely correlate with miR-29a and miR-29b expression in NPC cells, respectively. (i) The indirect effect of miR-29a to increase SPARC expression may be mediated by its unknown targets that affect cell survival, and subsequently SPARC could down-regulate the expression of COL3A1. (ii) COL3A1 is a direct target of miR-29b and affects the expression of the various ECM proteins, resulting in derepression of NPC cell migration and invasion.

For many tumor cells, increased levels of extracellular matrix proteins have been associated with an increased likelihood of clinical metastasis of multiple human solid tumors [35]. To confirm that the SPARC and COL3A1 protein is suppressed by miR-29a/b, we performed both miR-29a/b overexpression and knockdown experiments in S18 cells and examined SPARC and COL3A1 expression. As shown in Fig. 5A and B, SPARC was significantly down-regulated by cognate siRNAs and miR-29a in S18 cell lines (*p* < 0.05), whereas COL3A1 was more significantly down-regulated by their cognate siRNAs and miRNA-29b in S18 cells (*p* < 0.001). On the other hand, both SPARC and COL3A1 were increased in S18 cells that were transfected with the anti-miR-29a/b (*p* < 0.05). In fact, two recent studies suggested that SPARC modulates the expression of several ECM genes in a variety of cell types [36] and are good candidates as conditioners of the tumor matrix proteins [37]. These same genes are also predicted to be candidate miR-29a/b targets in our study. To further determine whether SPARC modulates expression of extracellular matrix proteins as an upstream regulator in NPC cells, we examined the effects of knock-down of the SPARC gene on COL3A1 proteins by their cognate siRNAs. As shown in Fig. 5C, SPARC siRNA-transfected S18 cells showed a greater reduction in SPARC expression compared with the control group, together with a significantly increased expression of COL3A1 (*p* < 0.01). However, after silencing the COL3A1 gene by transfection of cells with COL3A1 siRNA, the expression of SPARC proteins was not significantly altered compared with that in the control group (*p* > 0.05). Therefore, these data suggest that miR-29a/b increase NPC cell migration and invasion primarily by direct mir-29b targeting COL3A1 to down-regulate its cytoplasmic expression, together with indirectly affecting the stimulation of SPARC to COL3A1 by miRNA-29a.

### Amounts of SPARC protein correlate with NPC aggressiveness

**Fig 6:**
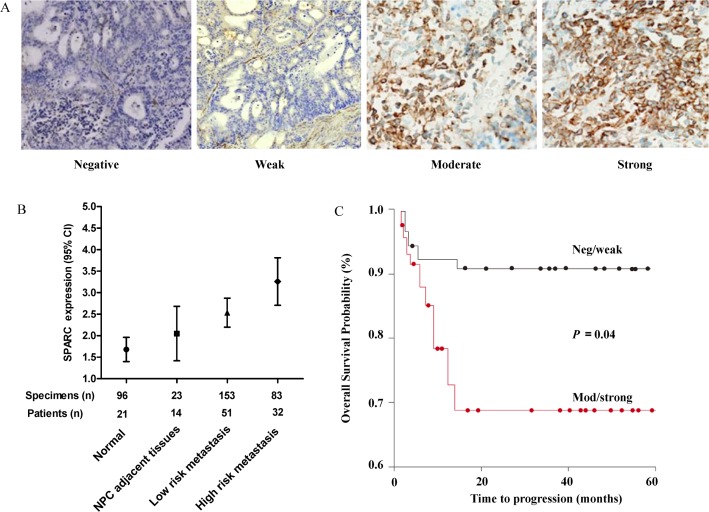
High SPARC expression correlates with shorter overall survival in NPC patients. (A) SPARC protein levels correlate with NPC aggressiveness. Representative tissue is stained with an antibody against NPC. Immunohistochemical stains show absent nuclear staining in normal samples (left, original magnification 100×), moderate and strong nuclear staining in NPC samples with low and high risk of metastasis (center right and right, respectively; original magnification 100×). Weak staining shown is a benign nasopharynx adjacent to NPC (center left, original magnification 100×). (B) Tissue analysis of SPARC expression for each tissue spot. The mean SPARC protein expression for the indicated NPC tissues is summarized using error bars with 95% confidence intervals, demonstrating a significantly lower score in NPC with a high risk of metastasis compared with non-malignant controls (Mann-Whitney test, two-tailed, *p* < 0.001). (C) The OS of patients with low SPARC expression levels was significantly higher than that of patients with high SPARC expression levels (log rank test, *p* = 0.04).

We evaluated the expression of SPARC protein in a wide range of NPC tissues (n = 320) to determine the extent of its expression in situ (Fig. 6A). When highly expressed, SPARC was distributed throughout the cytoplasm, as suggested previously [38]. The intensity of SPARC staining increased from benign (n = 96), non-disseminated NPC (n = 23), low metastatic/invasive cancer (n = 153), to high metastatic/invasive cancer (n = 83), with a respective median staining intensity of 1.2 (standard error (s.e.), 0.1, 95% confidence interval (CI), 1.3–1.6, 1.5 (s.e., 0.2, 95% CI, 1.4–2.2), 2.1 (s.e., 0.4, 95%CI, 1.7–2.5) and 2.8 (s.e., 0.3, 95% CI, 2.6–2.8), respectively (Fig. 6B). However, there was no significant difference in COL3A1 staining intensity between benign nasopharyngeal tissue and localized NPC cancer (data not shown). These findings suggested that, as NPC progresses, there is a trend towards increased expression of SPARC protein. They also suggested that SPARC concentrations might indicate the aggressive nature of an individual NPC, given that the highest expression was observed in highly metastatic NPC. Meanwhile, Kaplan–Meier analysis shows that individuals with clinically localized NPC with high expression of SPARC (moderate to strong staining) correlated with shorter overall survival in NPC patients (Fig. 6C). All of these findings suggest that SPARC may have an important role in promoting a more aggressive phenotype, similar to the behavior of NPC cells.

### Correlation of serum miR-29a/b expression with NPC patients survival

**Fig 7:**
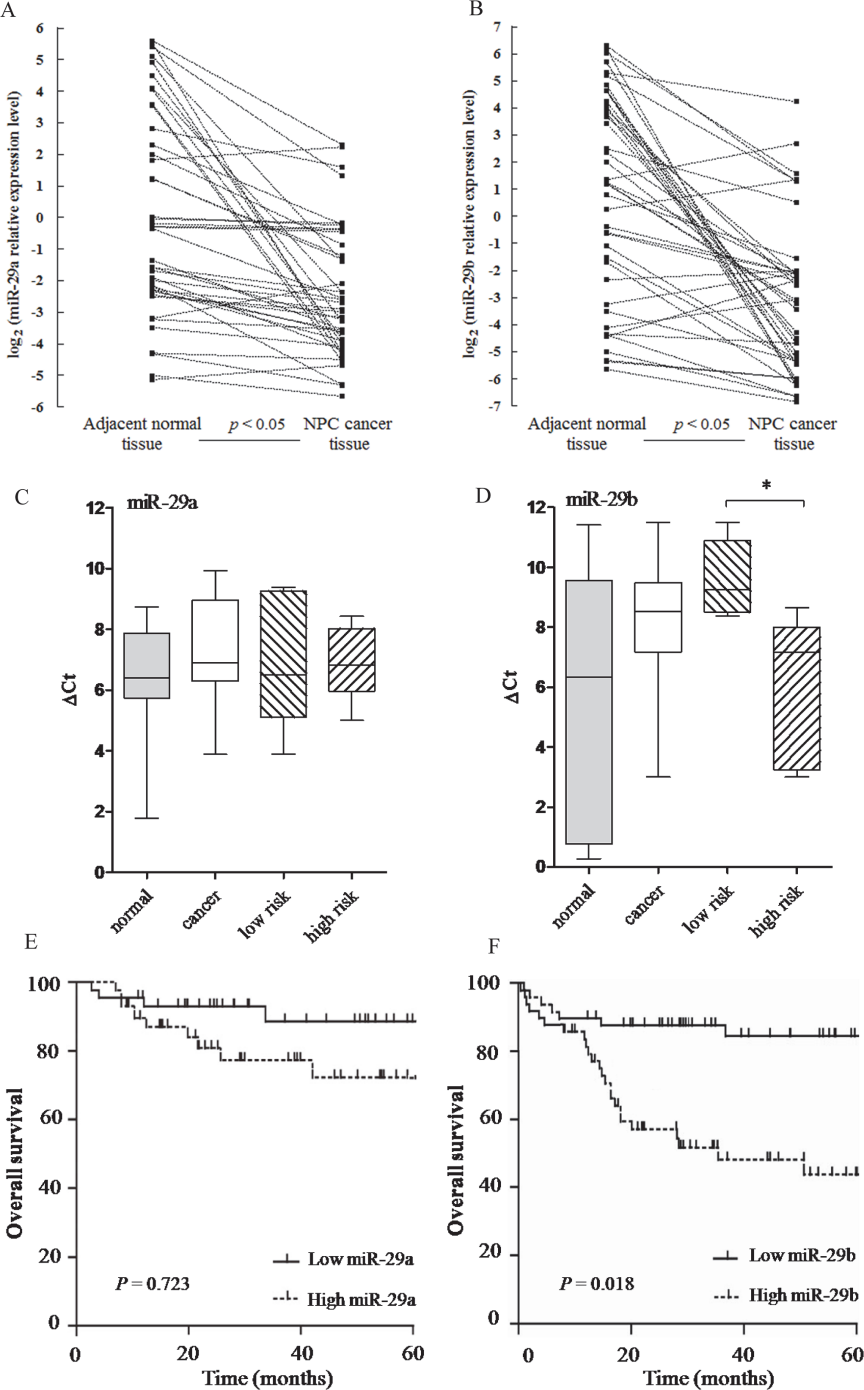
miR-29b is associated with specific risk groups and NPC patient survival. (A, B) Comparison of the miR-29a/b abundance in paired NPC tumors (42 NPC patients) and adjacent normal tissues (42 normal controls). The solid squares represent the relative expression level of miR-29a/b. The miR-29a/b abundance for each paired non-tumor and tumor tissues were separately shown in the left and right parts and connected by a dash line. (C, D) The expression levels of miR-29a/b in serum were quantified by real-time PCR in 83 patients with highly metastatic/invasive, 110 patients with low metastatic/non-invasive cancer and 65 healthy donors. (C) There was a small change in miR-29a expression in NPC patients and healthy donors, as well as patients with high risk for metastasis and the low-risk group. The formula used to calculate the relative Ct values was (ΔCt = assay Ct − control Ct). A higher ΔCt value indicates that the miRNA is less abundant in a sample. (D) miR-29b was significantly up-regulated in the NPC patients at high-risk for metastasis compared with the low-risk group. (E) Kaplan–Meier survival curves of NPC patients. No significant differences were observed in OS rates between patients with high and low miR-29a expression. (F) The 5-year overall survival rate of NPC patients with high serum miR-29b expression was significantly lower than that of those with low serum miR-29b expression (*p* < 0.001).

To gain insight into the biological role of miR-29a/b in human NPC development, we first determined the expression level of miR-29a/b in NPC tissues (n = 42) and normal adjacent tissues (n = 42) by real-time RT-PCR. Consistent with previous reports [10], miR-29a/b expression was significantly lower in cancer tissues than in normal adjacent tissues (*p* < 0.05;Fig. 7A and B). Furthermore, we analyzed the expression of miR-29a/b in 193 sera of NPC patients with a well-documented clinical course by real-time PCR. Neither miR-29a nor miR-29b showed a significant difference in expression between sera from NPC patients and sera from the control group (Fig. 7C and D). Because NPC is an aggressive cancer with a dismal outcome largely due to metastasis and invasion, we questioned whether miR-29a/b expression was associated with NPC migration and invasion. In NPC samples, we investigated for miR-29a/b expression in primary NPC sera from highly metastatic/invasive samples (M; n = 83) and from low metastatic/non-invasive samples (NM; n = 110) with respective evidence of metastasis at the time of surgery. We found that little change in miR-29a expression was observed in NPC patients and healthy donors, as well as in patients with a high or low risk for metastasis (Fig. 7C). Surprisingly, we identified that the relative Ct values of miR-29b could significantly discriminate the sera of M from NM samples based on real-time PCR (Fig. 7D). It appeared that miR-29b (value 4.60, ranging from 0.16 to 8.65) was most highly up-regulated in high-risk samples compared with low-risk samples for metastasis (value 9.75, ranging from 8.96 to 11.3) Therefore, up-regulation of miR-29b expression was significantly associated with tumor metastasis and invasion. Next, the overall survival rates of patients with high and low serum miR-29b expression were compared. The 5-year actuarial overall survival (OS) rates in NPC patients with high and low serum miR-29b expression levels were approximately 57.3% and 78.6%, respectively (Fig. 7F). The survival difference between these two groups was significant (*p* < 0.01). However, no significant differences were observed in OS rates between patients with high and low miR-29a expression (Fig. 7E). Taken together, these results suggest that higher expression levels of miR-29b were associated with worse survival and prognosis.

## Discussion

Bioinformatic algorithms have been constructed to predict microRNA gene targets by searching for sequence complementarity between the microRNA and the 3′-UTR of the gene target. More sophisticated methods for predicting targets of NPC miRNAs have demonstrated that paired expression profiling of mRNA and miRNAs could be utilized to precisely identify functional miRNA-target relationships without experimental validation and specific tissue expression [39]. Earlier studies have detected the down-regulation of miR-9, miR-34c-5p and miR-141 in NPC, and there was a distinct inverse relationship between the up-regulated gene CCNE2 and two miRNAs, miR-9 and miR-34c-5p [40, 41]. Furthermore, microarray analysis showed that miR-29a and miR-29b, which were predicted to target up-regulated genes within the interaction network, were under-expressed in NPC [27, 42]. However, these current analysis did not include estimates of false-positive rates which led a misunderstanding in functional correlation of miRNA. In the present study, we described an assembling approach that predicted 32 up-regulated and 139 down-regulated genes relative to nasopharyngeal tumorigenesis and provided computational and experimental evidence. Most of these genes were authentic targets, allowing us to explore fundamental questions regarding miRNA:target relationships in NPC. The accuracy and effectiveness of the screening in predicting microRNAs might be highly improved by inverse expression patterns in predicting miRNAs which would control dysregulated genes of NPC, in combination with the TargetScan, miRanda and miRbase Target programs. A complex interaction network has been developed after assembling the selected genetic data and a set of 9 miRNAs, which would harbor candidate tumor suppressor genes/oncogenes of NPC (Fig. 1A). These results could help identify the significant factors that brought about a complex balance in nasopharyngeal carcinogenesis. An interesting result produced by our prediction was that miR-29a and miR-29b jointly target genes were related to the extracellular matrix (Fig. 1B). Among these genes, COL4A1 and COL3A1, which encoded the collagen chain of basement membranes [43, 44], were found to be regulated by miR-29b [45]. However, NID1 encoded a protein that interacted with components of basement membranes [46], and this protein is also a target of miR-29b [47]. NID2 is a cell-adhesion protein that binds to collagens I/IV and laminin and may be involved in maintaining the structure of the basement membranes [48]. All of these genes encode some proteins that were important for the physiological or pathological formation of the extracellular matrix. Of note, miR-29c was down-regulated in NPC, which could up-regulate mRNA to encode matrix proteins. Most of the mir-29c-targeted genes were identified to encode extracellular matrix proteins that were associated with invasion and metastasis of NPC [10], which suggested that the miR-29 family may play roles in different phenotypes by regulating functionally related genes.

In our study, miR-29a/b over-expression resulted in decreased expression of SPARC and COL3A1 mRNA in the NPC cell line S18, which suggested that SPARC and COL3A1 could be targets of miR-29a and miR-29b respectively (Fig. 2B). In line with other reports, miR-29a and SPARC expression levels were inversely correlated in hepatocellular carcinoma cell lines, and over-expression of miR-29a resulted in a significant reduction in SPARC mRNA and protein of hepatocellular carcinoma and the trabecular meshwork [49, 50]. Meanwhile, SPARC down-regulation was observed when the human trabecular meshwork cells were transfected with an miR-29b mimic [20]. However, in miR-29a/b studies, the matching of the miR-29 target sites in the 3'-UTR of SPARC did not inhibit luciferase expression, which indicated that miR-29a did not directly target SPARC in NPC cells. On the other hand, miR-29a up-regulation significantly increased COL3A1 expression. This result may suggest that COL3A1 could be increased by reducing SPARC expression through miR-29a [51], while real-time PCR and dual luciferase reporter assays proved that COL3A1 was a direct target of miR-29b. In our preliminary investigations, we identified miR-29a/b was differentially expressed in a model of invasive NPC cells, and subsequent research found that miR-29a/b over-expression could significantly increase the mobility of the NPC cell line S18, which has a high metastatic capability. However, no difference in proliferation was observed when miR-29a/b was over-expressed (Fig. 3A). It was similar to previous studies demonstrated the increased expression of miR-29a could facilitate HepG2 cells migration [52]. This result was coupled with a slight decrease in the proportion of cells in the S phase (Fig. 3B), suggesting NPC cells incubated with miR-29a/b did not proliferate but rather maintained basal survival to withdrawing cells temporarily from the cell cycle in preparation for their migration and invasion. Previously, it has been shown that miR-29a over-expression could suppress the expression of tristetraprolin (TTP), a protein involved in the degradation of messenger RNAs with AU-rich 3'-UTRs, and lead to epithelial-to-mesenchymal transition and metastasis in cooperation with oncogenic Ras signaling [53]. In addition, MCF-7 cells transfected with pre-miR-29b had a greater migratory and invasive activity compared with the control group [54]. Conversely, miR-29a/b was shown to suppress tumor invasion and migration in human carcinoma cell lines [55, 56], which suggested that miR-29a/b have significant anti-invasive and anti-proliferative effects on cancer cells in vitro and function as anti-oncomirs. Other evidence has shown that enhanced miR-29b expression by transfection with pre-miR-29b could decrease PTEN expression and impair apoptosis, increasing tumor cell migration and invasion [54]. Furthermore, the decrease in endogenous miR-29c levels using a miR-29c inhibitor resulted in metastatic tumor invasion by up-regulating the extracellular matrix targets or related proteins [10], which was consistent with the data suggesting that increased expression of miR-29c impedes cell migration and invasion by targeting TIAM1 in both SUNE-1 and CNE-2 cells [57]. These studies have elicited some controversy because of the possibility that the role of the miR-29 family in tumors may be cell type- and context-dependent. Although the “seed regions” of the mature miR-29 family members are the same, their function may not be identical in NPC cells. Therefore, it is necessary to further explore whether the miR-29 family have same effect and mechanism on motility of NPC cells.

Earlier experiments have shown that SPARC effectively could inhibit cell spreading [58] and bind to specific components of the connective tissue ECM in a Ca2^+^-dependent manner [59]. However, the mechanism by which SPARC influencing cell migration is not known. One possibility was that the lack of SPARC caused alterations in the ECM with respect to structure and function and thus contributed to a decreasing/increasing in cell migration. We analyzed the 3'-UTR of SPARC mRNA from the NPC cell lines by RT-PCR and sequencing which did not find any mutations or deletions in the putative miR-29a binding site. In our experiments, we showed that miR-29a indirectly suppressed SPARC mRNA expression in the NPC cell lines (Fig. 2), and over-expression of miR-29a/b could significantly reduce the protein expression of SPARC and COL3A1 in S18 cells. Conversely, the inhibition of miR-29a/b increased the protein levels of SPARC and COL3A1 respectively (Fig. 5A and B). Furthermore, a 70% inhibition of SPARC with SPARC siRNA significantly up-regulated COL3A1 expression, while an 80% inhibition of COL3A1 with COL3A1 siRNA did not affect SPARC in S18 cells (Fig. 5C). Considering these observations, we have provided the first indication that there were two distinct regulatory pathways of miR-29a/b that would help to relieve the influence of ECM dysregulation on cellular physiology. miR-29a could indirectly silence SPARC through an unclear mechanism in which unknown factors may interfere with the interaction between miR-29a and SPARC mRNA. In addition, miR-29b directly suppressed COL3A1 to strengthen control of NPC cell migration and invasion (Fig. 5D). Moreover, SPARC, an upstream regulator of COL3A1, could inhibit COL3A1 expression in response to miR-29a stimulation, which we referred to as our integrated microRNA-gene network (Fig. 1), suggesting that our results could identify the reliability and accuracy of the gene-miRNA network.

NPC patients are commonly diagnosed in later stages because of vague early symptoms. There is little dispute that the early detection and treatment of NPC is important for increasing the likelihood of a cure for NPC patients. miRNAs are markers with clinical applicability for cancer diagnosis and prognosis, and they are relatively easier to be detected because of non-invasion and highly conserved [60]. miRNAs have been reported in the development and progression of many cancers, such as lung cancer, liver cancer and gastric cancer, and they are potential biomarkers for cancer diagnosis, prognosis, and personalized therapy [61–63]. In addition to the deregulation of cellular miRNAs in NPC [40], recent profiling studies using TaqMan low-density arrays and microarrays revealed the circulating expression levels of several miRNAs, including miR-17, miR-20a, miR-29c, and miR-223, as non-invasive biomarkers in NPC. The robust differential expression of miRNAs in blood-based samples is also to determine the functioning of key molecules in cell signal transduction and gene regulation network. Of note, there was also a higher level of SPARC expression in NPC that significantly correlated with progression and shorter overall survival (Fig. 6), suggesting that high SPARC expression in NPC tissues would down-regulate COL3A1 expression in the cytoplasm and affect downstream signaling pathways, which would increase migration and invasion in NPC cells. Thus, monitoring the amounts of SPARC protein in NPC specimens would provide additional prognostic information that could be no discernible with current clinical and pathology parameters alone. Unexpectedly, a significant increase of serum miR-29b was observed in NPC patients with the high risk group of metastasis rather than the low risk group (Fig. 7D). However, miR-29a expression was slightly up-regulated in NPC sera compared with the normal group (Fig. 7C), which showed that serum miR-29b was associated with metastasis risk in NPC oncogenesis, consistent with our results from the influence on S18 cells exerted by miR-29b. Therefore, we speculated that the miR-29b level in serum was associated with the pattern in the primary NPC tissue, in which, the degradation of cellular miR-29b in the biopsies with a high risk of metastasis may result in their up-regulation in sera of NPC patients.

In summary, miR-29a/b displayed aberrant expression in NPC tissue and serum samples, likely modulating SPARC and COL3A1 expression respectively, and contributing to migration and invasion. A practical application of our observations about serum miR-29b expression along with SPARC could be used as a potential predictor that may be more likely to respond to the NPC-mediated context. Further studies are needed to fully elucidate miRNA-involved physiological or pathological regulatory mechanisms, and it is of great significance to investigate miR-29a/b expression related to occurrence and development for the prevention and treatment of NPC.
